# Development and Validation of a Questionnaire to Assess Creative Potential Traits

**DOI:** 10.3389/fpsyg.2021.756079

**Published:** 2021-11-04

**Authors:** Julio C. Penagos-Corzo, Axel Saucedo

**Affiliations:** Department of Psychology, Universidad de las Américas Puebla, Cholula, Mexico

**Keywords:** creativity, risk-taking, rule-breaking, willingness to explore, transgression, personality

## Abstract

The aim of the study was to develop an inventory to assess traits of creative potential and to analyze its psychometric properties. Three dimensions that could be associated with creative potential were proposed: willingness to transgress, willingness to take on challenges, and willingness to explore. For this purpose 551 participants were chosen to respond to an inventory composed of 12 items: Traits of Creative Potential Questionnaire, (TCPQ-12) and other tests to determine concurrent validity. The correlations between these instruments were significant. In addition, the instrument showed adequate internal consistency (Ω = 0.813) and the exploratory factor analysis yielded salient factors coincident with the proposed dimensions. The confirmatory factor analysis indicated an optimal fit (CFI = 0.984, TLI = 0.979, GFI = 0.963, RMSEA = 0.035). with composite reliability (CR) values > 0.70 in two factors and in one was slightly lower (CR = 0.684). The average variance extracted (AVE) was > 0.5 in two factors and in one < 0.5. The study data allow to highlight that the instrument presented here is a concise instrument with adequate psychometric properties.

## Introduction

Creativity can be identified as a capacity that allows generating products that have originality and value (Runco and Jaeger, 2012; Diedrich et al., 2015; Corazza, 2016; Romo, 2019). There is evidence indicating that this capacity has biological (Sawyer, 2011; Penagos-Corzo, 2018; Deshayes et al., 2021), motivational (de Jesus et al., 2013; Liu et al., 2016; Taylor and Kaufman, 2021), affective (Baas et al., 2008, 2011; Mielniczuk and Laguna, 2020) and cognitive components (Benedek et al., 2014; Sampedro and Peña, 2019; Milicevic et al., 2020) that can enhance it. In addition to these components, some personality characteristics or traits have been associated with creativity practically since the contemporary beginning of its study (Guilford, 1950; MacKinnon, 1963) and in influential later works (Eysenck, 1993, 1994; MacKinnon, 1975/2017). More recent studies continue to yield evidence for the link between personality traits and creativity (George and Zhou, 2001; Furnham et al., 2008; Prabhu et al., 2008; Batey et al., 2010; Guo et al., 2021; Zhou, 2021). In fact, it is probably the individual’s unique personality that enables or hinders the development of creative ideas (Mumford and Gustafson, 1988). However, personality traits are relatively little addressed for the development of instruments related to the assessment of creative potential.

Openness to experience is a personality trait that can predict 10–50% of creativity (Sawyer, 2012). Its link to creativity is independent of the type of measure or domain of creativity (Hornberg and Reiter-Palmon, 2017). Some relevant characteristics of this trait are: preference for variety, challenge to authority, and curiosity (McCrae and Costa, 2008). The latter is not only related to creativity (Chang and Shih, 2019; Tsai and Zheng, 2021), but such relationship has a causal sense (Hagtvedt et al., 2019). In this sense, evidence indicates that curiosity positively predicts information seeking and negatively predicts intolerance of uncertainty (Jach and Smillie, 2021). This supports operational definitions of curiosity, which describe it as a preference for uncertainty (Jirout and Klahr, 2012). It can also be understood as a desire to discover information and experiences while motivating behavior (Hamilton, 2019). In fact it is an essential part of intrinsic motivation and can be considered as an innate need to explore (Grigorescu, 2020). Empirical data suggest that exploration is a vital component of creativity (Evans et al., 2021), as is intrinsic motivation (Amabile and Pratt, 2016). Data indicate that this is transmitted toward creativity in the form of an increased willingness and desire to take on challenges or risks (Dewett, 2007).

Conformity to social norms seems to be related to low creativity (Mu et al., 2015), and conversely nonconformity may be a predictive component of higher creativity (Fürst et al., 2016), especially impulsive nonconformity (Schuldberg, 1990). This is congruent with studies indicating that impulsivity and risk-taking are related to creativity (Xia et al., 2017). Higher risk takers score high on flexibility and originality (Glover and Sautter, 1977), which are characteristic variables of creativity. Consistent with the above, it has been suggested that people with high creativity possibly have more propensity toward risk (Sternberg and Lubart, 2002). In this sense, experimental studies indicate that environments that encourage risky decision making, as compared to environments that encourage safety-based decisions, produce more creative performances (Friedman and Förster, 2001). Indeed recreational and primarily social risk taking is linked to creative personality (Tyagi et al., 2017). When this risk-taking is assessed, what is observed are behaviors that generally transgress or violate norms or expectations (Highhouse et al., 2017). In children, transgressive play has been found to be related to better creative performances (Møller, 2015).

It is important to note that creativity is not something that happens in isolation. It is linked to the product and its social recognition (Romo, 2019). The social environment -or the field- somehow qualifies what is or is not creative (Csikszentmihalyi, 2013). However, the creative person is able to recognize opportunities in the environment and associated success (Chang and Chen, 2020). Given this interaction with the social environment, social skills are relevant so that the person who performs a creative act is not constrained by the impositions of the environment. In this sense, it has been observed that social skills are related to creativity (Lee et al., 2002; Hahn et al., 2011; Aydintan et al., 2014). Another variable that may be relevant in overcoming the pressures of the social environment is a willingness to challenge. Evidence indicates challenge seeking results in a good predictor of creative expression (Epstein and Phan, 2012).

Despite the above evidence regarding personality and creativity, there seem to be no quick and concise measures that assess traits predictive of creative potential. Some measures such as the Creative Personality Scale (CPS) (Gough, 1979) come close and even more recent versions can be found (Freiberg-Hoffmann et al., 2019). The CPS consists of a list of 30 adjectives. Eighteen are indicative of creativity and 12 are contraindicative. Examinees must select on a dichotomous scale (+ or −) whether the item describes them. The score is obtained by adding the selected indicative adjectives and subtracting the chosen contraindicative adjectives. The CPS in its first version (Gough and Heilbrun, 1965/1983) obtained an internal consistency of 0.63. It was subsequently tested in four samples (Gough, 1979). The internal consistency of the instrument in these samples ranged from an alpha of 0.73 to 0.81 and convergent validity indices ranged from 0.14 to 0.40. More recent work reports an alpha of 0.85, but with a weak convergent validity correlation of 0.20 and a unifactorial structure (Freiberg-Hoffmann et al., 2019). Other studies indicate alphas between 0.59 and 0.64 and a culture-dependent multifactor structure (Luescher et al., 2019). In previous paragraphs it was indicated that there was evidence that personality variables linked to exploration (Evans et al., 2021), transgression (Møller, 2015; Fürst et al., 2016) and risk-taking or challenge (Dewett, 2007; Epstein and Phan, 2012) predict creative performances. Therefore the aim of the present study is to evaluate the psychometric properties of a trait inventory of creative potential based on these three dimensions.

## Materials and Methods

### Participants

The sample consisted of 551 Mexican participants, of whom 356 were women and 195 men. The mean age was 34.3 years (SD = 16.2). The schooling of the women was as follows: 51.97% were college students, with an average age of 20.6 years (SD = 5.6), 40.45% were graduates and/or postgraduate students, with an average age of 46.2 years (SD = 11.1) and finally 7.58% had a high school diploma, with an average age of 47.5 years (SD = 13.7). The schooling of the males was as follows: 40.51% had undergraduate studies, with an average age of 21.2 years (SD = 5.5), 53.85% were graduates and/or postgraduate students, with an average age of 47.9 years (SD = 12.1) and finally 5.64% had only studied up to high school, with an average age of 47.5 years (SD = 17.3). The sample was split into two, one for exploratory factor analysis (subsample A [SSA]) and one for confirmatory factor analysis (subsample B [SSB]). The SSA (*N* = 276) consisted of 162 women and 114 men. The SSB (*N* = 275) consisted of 194 females and 81 males. All subjects gave written informed consent in accordance with the Declaration of Helsinki (World Medical Association, 2013). This study was reviewed and approved by the Ethics Committee of the Universidad de las Américas Puebla.

Undergraduate students were invited to participate through an invitation made in various classes and also through the official social networks of the university’s psychology department. An online invitation was also made to the adult public, through a mailing list of professionals. Not having completed high school and being under 18 years of age were considered as exclusion criteria. The questionnaires of participants who responded to all the instruments in a period of less than four minutes were also excluded, as this was considered an unreasoned response strategy and probably random, due to the impossibility of having read all the items, evaluated and selected the answer in this time. The application also incorporated four items to evaluate social desirability and random responses. If participants answered positively to one of these items, their questionnaires were not taken into account. An example of an item to assess whether the person was responding randomly was: “I forget how to spell my name.” An example to assess social desirability is the item: “I have never told a single lie in my life.” 22 participants were discarded because they met any of the exclusion criteria. The inclusion criterion was that they were university students or working adults.

### Instruments

*Traits of Creative Potential Questionnaire* (TCPQ-12). This questionnaire was developed in the present study and has twelve items created based on three dimensions: Exploration, Transgression, Challenge. The response options are by means of a seven-point scale, where 1 = does not look like me, 7 = looks like me.

*Big 5: Openness to experience factor* (Benet-Martínez and John, 1998). This instrument assesses five personality factors. The validation for Mexican population (Zamorano et al., 2014) was used, in which an adequate consistency was reported (α = 0.72). Only the factor of openness to experience was applied, consisting of 10 items (α = 0.77).

*Domain Specific Risk Taking (Dospert): Social and Recreational Factors* (Blais and Weber, 2006). The 30-item Dospert scale (α = 0.71 to 0.86) assesses domain specific risk taking. The subdomain of risk taking in social (6 items) and recreational (6 items) areas was used. This test is answered by choosing a number between 1 (Extremely unlikely) and 7 (Extremely likely) according to the probability that a behavior will be committed. Higher scores indicate greater risk taking.

*Political Skill Inventory (PSI): Social Astuteness Factor* (Ferris et al., 2005). This 18-item instrument (α = 0.89) assesses political skill. The social astuteness factor (5 items) was taken, which assesses the ability to observe and evaluate others, as well as adjust behavior in a wide range of social contexts (α = 0.80). This instrument is answered from 1 to 7 where 1 is “strongly disagree” and 7 is “strongly agree.” Higher scores indicate greater social astuteness.

*The Cognitive Flexibility Inventory* (CFI) (Dennis and Vander Wal, 2010). The Cognitive Flexibility Inventory contains 20 items (α = 0.90) measures an individual’s ability to change cognitive sets and adapt to changing stimuli in the environment. It is composed of two subscales: a) Alternatives (13 items) (α = 0.91) and b) Control (7 items) (α = 0.86). This inventory is answered by rating from 1 to 7 scores, where 1 is “I strongly disagree” and 7 is “I strongly agree.” Higher scores represent greater flexibility.

*Work Preferences Inventory* (WPI) (Amabile et al., 1994). This instrument evaluates intrinsic and extrinsic motivation. A version that was validated in a Mexican sample was used (Penagos-Corzo et al., 2017). Only the intrinsic motivation factor (α = 0.82) was applied, divided into two subscales: (a) Joy subscale with 7 items (WPI-G) (α = 0.65) and (b) Challenge subscale with 5 items (WPI-D) (α = 0.65). This instrument has 4 response options: from “Never or almost never true in my case,” to “Always or almost always true in my case.” Higher scores represent an individual with high intrinsic motivation.

*Kaufman Domains Of Creativity Scale* (K-DOCS) (Kaufman, 2012). The K-DOCS is an inventory that measures creativity from self-assessments on reported behaviors, focusing on 5 specific domains: Self/Everyday (α = 0.86), Scholarly (α = 0.86), Performance (α = 0.87), Mechanical/scientific (α = 0.86), and Artistic (α = 0.83). Response options range from 1 to 5 where 1 is “much less creative” and 5 is “much more creative.” Higher scores indicate greater creativity.

### Procedure

**FIGURE 1:**
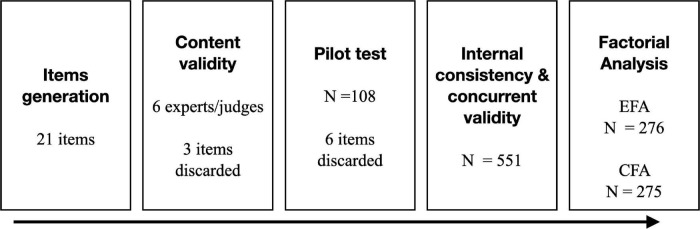
Steps from item generation to analysis to factor analysis.

The psychometric study carried out started with the initial development of items, which were evaluated by a group of experts, then tested in a pilot study and finally the resulting items were applied to the general sample for reliability and validity analyses. The graphical abstract shows this process (Figure 1).

#### Item Development

For the generation of the items, the recommendations indicated by Boateng et al. (2018) were taken into account, placing special emphasis on the deductive or logical partitioning method. This is done through literature review and evaluation of existing indicators of the domain to be measured. The items were developed in consideration of the findings of personality variables that have effects on creativity: Willingness to explore (Evans et al., 2021), transgressing norms or rules (Møller, 2015; Fürst et al., 2016), Willingness to challenge (Dewett, 2007; Epstein and Phan, 2012). Based on the above, the authors developed and determined 21 items that were intended to measure the three dimensions of the instrument. Seven items were created for each dimension. These items were the ones that the experts evaluated to determine the content validity.

#### Content Validity

For content validity, these items were given to six creativity experts. All experts taught university courses related to the research topic of the present study and/or had publications on the topic. These experts were asked to evaluate the importance of the items using a three-level criterion: (a) the item is essential, (b) the item is useful but not essential, and (c) the item is not essential. To determine the items with content validity, Lawshe’s (1975) content validity ratio (CVR) formula was applied, which states that $CVR=\frac{ne-\frac{\text{N}}{2}}{\frac{\text{N}}{2}},$ where ne = number of experts who evaluated the item as essential and *N* = total number of experts. The selection of the items was made based on the criteria of Tristaìn-López (2008): those items whose formula score was greater than 0.58 were eligible. This analysis resulted in 18 items.

#### Pilot Study

The 18 items were tested on a pilot sample of 108 participants. The sample was selected through an invitation made in social media of the Psychology Department of the University and in professional social networks. 59 females with a mean age of 34.05 (SD = 12.24) and 49 males with a mean age of 31.57 (13.43), made up this sample. 74.58% of the females were graduate or postgraduate students, while the remaining percentage were undergraduate students. 57.14% of the males were graduate or postgraduate students, while undergraduate students made up the remaining percentage. This sample was divided into two groups to test the discrimination capability of the items: One with the 27% of participants who obtained the highest scores and another with the 27% of participants who obtained the lowest scores. With these two groups, the discrimination capability of the items was analyzed through a t-test, comparing the high vs. low group in each item (Penagos-Corzo et al., 2019). Four items that did not have significant differences at a confidence level of 0.01 were discarded. Two additional items were eliminated because they were considered clearly repetitive and a correlation analysis confirmed this (0.971–0.979).

#### Application of Instruments for Reliability and Validity Analysis

After this analysis, the TCPQ was administered online, along with the other instruments, to the sample of 551 participants. The other instruments were selected to assess concurrent validity. Positive relationships were hypothesized between each of the scales and the salient factors of the TCPQ. Specifically, K-Docs was expected to have the highest correlations with the instrument under development. If the transgression scale was confirmed, it was expected to correlate with DOSPERT and WPI. On the other hand, if the Exploration scale were confirmed, the highest correlations would be expected with the Openness factor of the BigFive, with the intrinsic motivation subscales of the WPI, as well as with the CFI, specifically with the alternatives subscale. Whereas for the possible Challenge dimension, correlations were expected with Social Astuteness, the intrinsic motivation subscales of the WPI and the Openness factor of the BigFive.

### Statistical Analysis

Statistical analyses were performed in SPSS 24, AMOS 24 and Jamovi 2.0. An exploratory factor analysis (EFA) of the scale was performed, using principal axis factoring and promax rotation due to the conceptual consideration that the underlying factors may be correlated. Confirmatory factor analysis was assessed with the comparative fit index (CFI) the Tucker-Lewis index (TLI) and with the root mean square error of approximation (RMSEA). There is general agreement to use cut-off points of 0.95 for the CFI and TLI fit indices to consider an optimal fit, as well as values lower than 0.06 for the RMSEA (Hu and Bentler, 1999; Barrett, 2007). However, it has been suggested that > 0.97 for CFI and TLI, as well as < 0.05 for RMSEA are more advisable (Schermelleh-Engel et al., 2003), so the present study will use this recommendation. For GFI, a cut-off point > 0.95 will be used, for AGFI > 0.90, for PNFI > 0.60 and for SRMR < 0.05 which are also the recommended cut-off point of Schermelleh-Engel et al. (2003). For PCFI, the cut-off point > 0.6 will be used (Marsh and Grayson, 1995). Internal consistency was evaluated using McDonald’s omega. Finally, correlations between instruments and temporal stability were determined by Pearson correlations.

## Results

### Exploratory Factor Analysis

**FIGURE 2:**
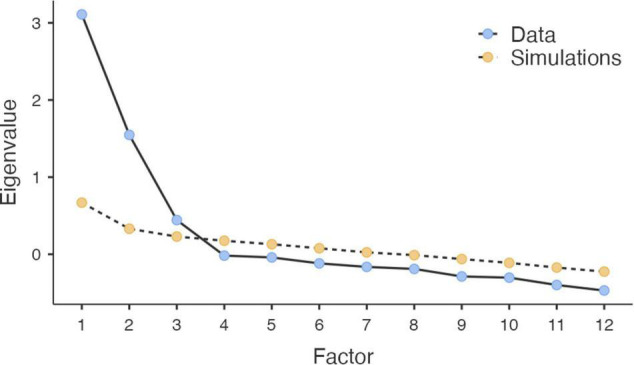
Scree plot with data and simulations (parallel analyses).

The exploratory factor analysis with the SSA sample indicated a KMO of 0.823, and Bartlett’s test of sphericity was significant (X266 = 1062.086, *p* < 0.001), indicating that it was pertinent to perform the factor analysis. The extraction criterion was an eigenvalue > 1.0 (Kaiser, 1960). In addition, a parallel analysis was performed (Horn, 1965). The AFE yielded a three-factor structure (Table 1), both with the Kaiser criteria (eigenvalue > 1.0) and with the parallel analysis (Figure 2). The first factor yielded an eigenvalue of 3.825 and explained 31.88% of the total variance, while the second factor had an eigenvalue of 2.315 and explained 19.29% of the total variance, and the third factor had an eigenvalue of 1.180 and explained 9.83% of the total variance. The three factors explained 61% of the total variance.

**TABLE 1:** Factor loadings.

**Items**	**F1**	**F2**	**F3**
i01 I have broken rules or regulations in order to implement my ideas or proposals (*Me he saltado normas o reglas para lograr implementar ideas o propuestas mías*)	**0.870**	0.183	–0.122
i04 I skip procedures or rules because of the excitement of doing some work. (*Me salto procedimientos o normas por la emoción de realizar algún trabajo*)	**0.763**	–0.095	0.066
i07 I have developed ideas that involve violation of certain rules. (*He desarrollado ideas que involucran violación de ciertas normas*)	**0.724**	0.034	–0.083
i10 I don’t mind breaking the rules to get a better job. (*No me importa romper las reglas para lograr un mejor trabajo*)	**0.655**	–0.106	0.105
i02 I think I dare more than the average person. (*Creo que me atrevo más que el común de las personas*)	0.037	**0.727**	0.001
i05 I have created original and positive things that are recognized by many people. (*He creado cosas originales y positivas que son reconocidas por bastantes personas*)	–0.018	**0.706**	–0.090
i08 In my projects I am definitely riskier than others. (*En mis proyectos definitivamente soy más arriesgado/a que los demás*)	0.003	**0.673**	0.118
i11 I get my ideas to have an impact on others. (*Logro que mis ideas tengan un impacto en los demás*)	–0.040	**0.630**	0.126
i03 I like to explore what is around me, in my environment. (*Me gusta explorar lo que hay alrededor, en mi entorno*)	0.016	0.024	**0.653**
i06 I tend to see things from different perspectives. (*Tiendo a ver las cosas desde distintas perspectivas*)	0.032	0.022	**0.634**
i09 Just for pleasure, I strive to find out how things work. (*Sólo por gusto, me esfuerzo en descubrir cómo funcionan las cosas*)	0.112	0.045	**0.516**
i12 Faced with a difficult choice, I follow my intuition. (*Ante una elección difícil, sigo mi intuición*)	–0.096	–0.014	**0.502**

*Salient and highest loadings per item in bold.*

### Confirmatory Factor Analysis

**FIGURE 3:**
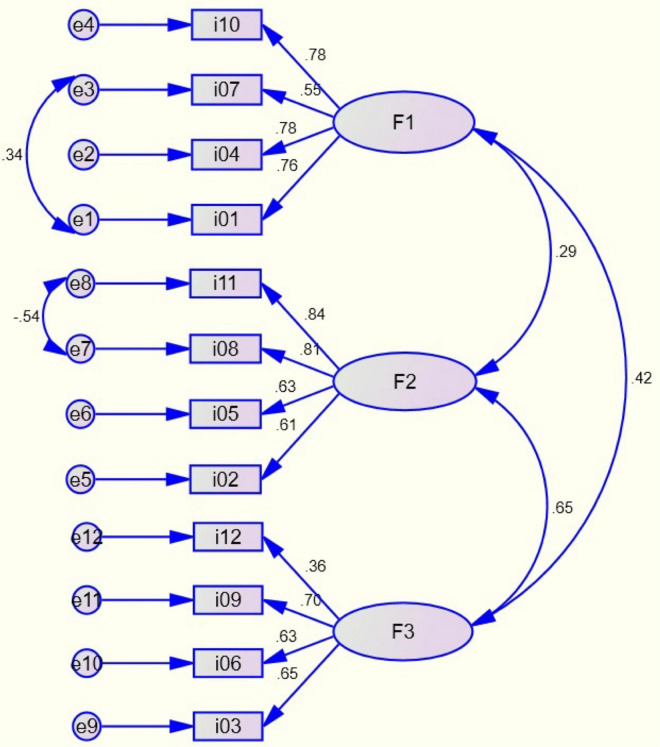
Path analysis diagram.

A confirmatory factor analysis was performed on the SSB sample using the maximum likelihood estimation method. The model fit indices suggested an adequate fit χ^2^(49) = 65.02, *p* = 0.062 (χ^2^/DF = 1.327), with optimal levels CFI = 0.984, TLI = 0.979 and RMSEA = 0.0347 (90% confidence interval, 0.00 Lower, 0.0554 Upper. pClose = 0.881). As shown in Figure 3, two pairs of items were co-varied, one for F1 (i01- i07) and one for F2 (i08 - i11). The goodness of fit index, parsimony and root mean squared residual also showed acceptable levels (GFI = 0.963, AGFI = 0.942, PNFI = 0.698, PCFI = 0.731, SRMR = 0.0435). The composite reliability data were > 0.70 for F1 (0.811) and F2 (0.816). For F3 the value was close to the cut-off point (0.684). The average variance extracted was also acceptable (>0.5) for F1 (0.522) and F2 (0.531), but for F3 the value was below the cut-off point (0.362). The discriminant and convergent validity analyses are presented in Table 2.

**TABLE 2:** Discriminant and convergent validity.

	**CR**	**AVE**	**√AVE**	**MSV**	**MaxR(H)**
F2	0.816	0.531	0.729	0.428	0.846
F1	0.811	0.522	0.723	0.174	0.829
F3	0.684	0.362	0.602	0.428	0.717

*CR, composite reliability; AVE, average variance extracted; √AVE, root square of the AVE; MSV, maximum shared variance; MaxR(H), maximum reliability.*

The discriminant validity analysis indicates that the AVE of F1 and F2 have higher values than the MSV. However, the AVE of F3 is lower than the MSV and also the square root of the AVE of F3 is lower than the value of the correlations with another factor (Table 2). In addition, the AVE values obtained indicate for F1 and F2 adequate convergent validity, but F3, as already noted, has a value <0.05.

#### Analysis of Invariance

The structure of the TCPQ was analyzed between both sexes (M1). The data indicate an adequate fit (CFI = 0.954, GFI = 0.924, RMSEA = 0.042) in this analysis. Subsequently, this analysis was performed in a fully constrained model (M2). With this model the fit indices were also appropriate (CFI = 0.957, GFI = 0.920, RMSEA = 0.039). The differences in chi-square between both models indicate invariance (*X*^2^_*M1*_ = 145.5, *df*_*M1*_ = 98, *X*^2^_*M2*_ = 154.8, *df*_*M2*_ = 110, *p* = 0.677).

### Concurrent Validity

Each of the resulting factors and the total TCPQ were correlated with the other scales applied to the total sample (*N* = 551). As can be seen in Table 3, in general the correlations are moderate and were significant. As expected for concurrent validity, the highest correlations are with K-Docs, BigFive openness and WPI intrinsic motivation. The predictions about the relationship between the transgression factor of the TCPQ and the DOSPERT, as well as with the challenge subscale of the WPI, are confirmed. While for the challenge factor of the TCPQ, the predictions of relationship with the social astuteness of the PSI, the intrinsic motivation subscales of the WPI and the openness factor of the BigFive are also confirmed. Similarly, the relationship of the exploration factor of the TCPQ with the openness factor of the BigFive and with the intrinsic motivation subscales of the WPI is also confirmed.

**TABLE 3:** Pearson’s correlations amongst tests and factors.

**Factors/Scales**	**1**	**2**	**3**	**4**	**5**	**6**	**7**	**8**	**9**	**10**	**11**	**12**	**13**	**14**	**15**	**16**	**17**
1 TCPQ-Tot																	
2 TCPQ -Challenge	0.717***																
3 TCPQ -Exploration	0.689***	0.466***															
4 TCPQ -Transgression	0.72***	0.175***	0.243***														
5 K-DOCS-Tot	0.555***	0.506***	0.486***	0.256***													
6 K-Docs-Self/Everyday	0.477***	0.504***	0.486***	0.134**	0.637***												
7 K-Docs-Scholarly	0.524***	0.509***	0.444***	0.233***	0.746***	0.563***											
8 K-Docs-Performance	0.311***	0.268***	0.227***	0.181***	0.772***	0.332***	0.439***										
9 K-Docs-Mechanical/Scientific	0.402***	0.314***	0.335***	0.237***	0.689***	0.261***	0.392***	0.397***									
10 K-Docs-Artistic	0.385***	0.341***	0.371***	0.158***	0.821***	0.455***	0.508***	0.609***	0.479***								
11 CFI: Alternatives	0.336***	0.376***	0.426***	0.024	0.425***	0.556***	0.447***	0.235***	0.199***	0.301***							
12 CFI: Control	–0.045	−0.16***	–0.02	0.085*	–0.06	−0.15***	–0.03	0.025	−0.11**	–0.01	−0.18***						
13 WPI: Challenge	0.515***	0.43***	0.459***	0.292***	0.476***	0.404***	0.47***	0.242***	0.431***	0.293***	0.361***	−0.09*					
14 WPI: Enjoyment	0.37***	0.339***	0.45***	0.096*	0.395***	0.508***	0.404***	0.199***	0.145***	0.313***	0.439***	0.013	0.374***				
15 Dospert: Social	0.325***	0.232***	0.358***	0.146***	0.306***	0.306***	0.34***	0.162***	0.135**	0.234***	0.327***	0.025	0.327***	0.288***			
16 Dospert: Recreational	0.351***	0.146***	0.302***	0.335***	0.326***	0.229***	0.25***	0.196***	0.302***	0.233***	0.124**	0.054	0.363***	0.184***	0.325***		
17 PSI: Social Astuteness	0.445***	0.46***	0.39***	0.174***	0.454***	0.518***	0.449***	0.295***	0.185***	0.353***	0.612***	−0.12**	0.381***	0.418***	0.343***	0.214***	
18 Big 5: Openness	0.557***	0.547***	0.563***	0.198***	0.555***	0.49***	0.51***	0.341***	0.341***	0.446***	0.451***	0.061	0.452***	0.41***	0.3***	0.198***	0.471***

*p < 0.05, **p < 0.01, ***p < 0.001.

### Reliability

#### Internal Consistency

The results of the reliability analysis by means of the omega coefficient indicate acceptable levels for the total instrument (Ω = 0.813), as for F1 (Ω = 0.833), F2 (Ω = 0.797) and F3 (Ω = 0.683). The internal consistency analysis was performed with the total sample (*N* = 551).

#### Temporal Stability

The temporal stability analysis was conducted with a combination of the participants from the original sample who agreed to answer the questionnaire again (*n* = 188) and an additional sample (*n* = 55) recruited to increase the sample size (*N* = 243). The retest (post measurement) was done after a period of 30–50 days had elapsed. Correlation analysis indicated adequate temporal stability for each factor: F1 (*r* = 0.803; *p* < 0.001), F2 (*r* = 0.759; *p* < 0.001), and F3 (*r* = 0.841; *p* < 0.001), as well as for the instrument total (*r* = 0.861; *p* < 0.001).

### Normative Data

The sample data (*N* = 551) indicate a mean of 53.7 for the total TCPQ and a standard deviation of 12.0. The factors obtained the following values: F1 (M = 12.4, SD = 6.37), F2 (M = 19.4, SD = 5.58), F3 (M = 22, SD = 4.32).

## Discussion

The analysis of the psychometric properties of the instrument analyzed indicates both a good fit to the model and acceptable reliability. The salient factors, transgression (F1), challenge (F2) and exploration (F3), correlate moderately and significantly with the scales used, which can be considered as evidence of validity.

The factor named transgression, referring to the violation of norms, maintains a good relationship with the total of the instrument and has the highest explained variance. A meta-analytic work had already reported that creative people question norms (Feist, 1998). This would also imply a probable relationship with intrinsic motivation, in the absence of explicit extrinsic rewards derived from the transgression of norms. This is relatively confirmed by our findings, since the highest F1 correlations are with the Challenge factor of the WPI and the Recreational factor of the Dospert. More recently it has also been pointed out that this norm questioning is part of a group of personality variables that correlate positively with creativity (Feist, 2019). Indeed, creative people may be compelled to violate norms in order to promote change (Bonetto et al., 2021). However, there does not seem to be much evidence in this regard. On an anecdotal level, examples can be pointed out in the case of art. Great creators, for example Picasso, Stravinsky, or Stein broke - transgressed - the canons of their discipline. It is likely that the lack of evidence is due to the fact that transgression is adaptive and not socially harmful. This may yield contradictory data, since rule-breaking is usually posed in an antisocial sense and it is likely that the creative person does not transgress these rules. In this sense, there is evidence indicating that rules also have a positive relationship with creativity (Du et al., 2021), because they can promote positive expectations for creative behavior (Liu Y. et al., 2021).

The challenge factor confirms that willingness to take risks is a variable linked to creativity, as some evidence has reported (Dewett, 2006). F2 may imply that the intellectual stimulation associated with risk taking allows considering alternative perspectives (Liu H. et al., 2021). It could also be explained from an association with the flow state noted by Csikszentmihalyi (2013). This state occurs in a positive relationship between task challenges and task skills (Hamari et al., 2016) and there is evidence about this state being linked to creativity (MacDonald et al., 2006; Dan, 2021). The factor includes items that not only refer to risk-taking but also to one’s own perception of recognition and social impact. The creative person willing to challenge may be aware of a socially recognized achievement and the resulting organization of the items indicates this. This would indicate a form of self-efficacy. In fact, it has been reported that when risk willingness is high there is an indirect effect on creativity through creative self-efficacy (Zhang et al., 2015). Furthermore, the correlation of F2 with social astuteness measured with the PSI suggests that social skills may be relevant for taking on challenges related to creative potential.

The desire to know or curiosity is a motivational characteristic and is related to the willingness to explore (Schutte and Malouff, 2020). The items that constitute F3, have this characteristic, and also integrate characteristics of openness and flexibility. Thus, the salient factor supports other findings that point to openness as a preference for exploration and variety, from an empirical point of view (Matz, 2021). Furthermore, the positive correlations between F3, with the openness and BigFive factor and with the intrinsic motivation subscales of the WPI support this notion. On the other hand, the moderate correlation with the “Alternatives” subscale of the CFI confirms the relationship expectations raised in the procedure. At the same time, it indicates the feasibility of establishing cognitive correlates, in this case flexibility, with personality traits such as openness. The above adds to other similar findings (Murdock et al., 2013).

The convergent and discriminant validity analyses yielded some values that may cause concern. F3, showed a value of less than 0.5 in AVE, while in composite reliability it also obtained a value slightly lower than 0.70. It has been suggested that a value below 0.5 may be acceptable because AVE is a conservative measure (Fornell and Larcker, 1981). Values between 0.6 and 0.7 of CR may also be acceptable when other indicators of model validity are good (Hair et al., 2014), as is the case in the present study. However, the values found question the full validity of the model, despite optimal values in other CFA indices and other reported validity evidence. Therefore, it is suggested that in future studies the number of items in F3 should be increased and it is probably advisable to eliminate item 12, which has the lowest factor loadings. In our analyses this adjustment did not yield any significant modification, but the inclusion of other items or the replacement of item 12 may have a significant effect.

A finding worth highlighting is that the correlations of the TCPQ-12 with convergent validity measures are similar to the correlations of the K-Docs with the same measures. This, coupled with the correlation between TCPQ-12 and K-Docs, gives greater consistency to the validity evidence found.

### Scope and Limitations

The present study presents some evidence of initial validity, but its relationship with creative potential performance remains to be empirically determined. Since one of the most commonly used measures to evaluate creative potential is divergent thinking, it is suggested that this relationship be analyzed in future studies. For example, correlational studies can be done with the CREA (Corbalá-Berná et al., 2016), or with the Torrance Tests of Creative Thinking (Torrance and Presbury, 1984). This will make it possible to address the predictive validity of the TCPQ-12 in greater depth. For this purpose, it is also suggested to conduct studies in which, sometime after applying the TCPQ-12, the participant’s creative performance in specific tasks or domains is evaluated. The present research was done during the COVID-19 pandemic, which prevented and still prevents us from assessing the participants directly as the convergent thinking tests mentioned above would demand. It is also important to note that the present instrument is not intended to assess a creative way of acting or creative characteristics, as could be done with the rCAB (Runco, 2011) but traits that are associated with creative potential. In addition, examining the invariance of the instrument would help to better determine its scope and limitations. Finally, F1 was developed as a form of adaptive or positive transgression. For this reason we did not propose to study its relationship with any explicit measure of transgression, as they are typically associated with antisocial behavior. However, perhaps future studies could address this, especially for malevolent creativity (Hao et al., 2016).

### Conclusion

In addition to achieving a concise instrument with adequate psychometric properties for a first version of the inventory, the present study provides empirical evidence on individual traits and dispositions related to creative potential. Presenting three of these dispositions as a model that contributes to explaining part of the creative potential is a novel proposal. Exploration orientation, flexible, self-motivated, willing to take on challenges and to positively transgress social norms seem to be relevant to creative potential. The items that make up the salient factors allow us to make the above point. The TCPQ-12 seeks to evaluate only some traits in an integrated way, but looking for a translational sense. For example, the TCPQ-12 can be used in organizational settings as a possible predictor of creative potential at work.

Finally, like any psychological variable, creativity happens in a multifactorial context. Predicting creative potential is a challenge with a long way to go. Several variables mediate this process, for example affective or emotional variables (Baas et al., 2008; Ivcevic and Brackett, 2015), cognitive (Benedek et al., 2014), and of course environmental characteristics (Chi et al., 2020), to name a few. However, having forms of measurement such as the one presented here help to address this challenge.

## Data Availability Statement

The datasets presented in this article are not readily available due to protection of participant privacy. Requests to access the datasets should be directed to JP-C, julioc.penagos@udlap.mx.

## Ethics Statement

The studies involving human participants were reviewed and approved by Ethics Committee at Universidad de las Américas Puebla. The patients/participants provided their written informed consent to participate in this study.

## Author Contributions

Both authors contributed, to varying degrees, in each of the following: conception of the study, methodology and formal analysis, writing of the manuscript, review of form and content, and approval of the final version of the manuscript. JP-C was in charge of the project administration and supervision.

## Conflict of Interest

The authors declare that the research was conducted in the absence of any commercial or financial relationships that could be construed as a potential conflict of interest.

## Publisher’s Note

All claims expressed in this article are solely those of the authors and do not necessarily represent those of their affiliated organizations, or those of the publisher, the editors and the reviewers. Any product that may be evaluated in this article, or claim that may be made by its manufacturer, is not guaranteed or endorsed by the publisher.
